# Interaction Between the Matrix Protein and the Polymerase Complex of Respiratory Syncytial Virus

**DOI:** 10.3390/v16121881

**Published:** 2024-12-04

**Authors:** Elliot B. Atchison, Sarah N. Croft, Cynthia Mathew, Daniel W. Brookes, Matthew Coates, Kazuhiro Ito, Reena Ghildyal

**Affiliations:** 1 Faculty of Science and Technology, University of Canberra, Canberra, ACT 2617, Australia; elliot.atchison@gmail.com (E.B.A.); sarah.croft@anu.edu.au (S.N.C.); cynthia.mathew@act.gov.au (C.M.); 2 Pulmocide Ltd., London WC2A 1AP, UK; dwbrookes@gmail.com (D.W.B.); mattcoates84@googlemail.com (M.C.); k.ito@imperial.ac.uk (K.I.); 3 National Heart and Lung Institute, Imperial College London, London SW3 6LY, UK

**Keywords:** respiratory syncytial virus, L protein, M protein, air liquid interface culture, bronchial epithelium, nuclear translocation

## Abstract

The global burden of respiratory syncytial virus (RSV) and severe associated disease is prodigious. RSV-specific vaccines have been launched recently but there is no antiviral medicine commercially available. RSV polymerase (L) protein is one of the promising antiviral targets, along with fusion and nucleocapsid proteins. During medicinal chemistry campaigns, two potent L-protein inhibitors (PC786 and PC751) were identified. Both compounds inhibited the RSV A/B-induced cytopathic effect in HEp-2 cells equally, but PC786 was more potent than PC751 in bronchial epithelial cells. Repeated treatment with escalating concentrations on RSV A2-infected HEp-2 cells revealed both inhibitors led to a Y1631H mutation in the L protein, but only PC786 induced a mutation in the M protein (V153A). By L protein fragment and M protein binding analysis, we showed that the M protein interacts with the 1392–1735 amino acid region of the L protein, where PC786 potentially binds. In addition, PC786 treatment or PC786-induced mutant RSV was found to increase M-protein nuclear localisation later in infection, concomitant with delayed fusion protein localisation at the budding viral filaments. As M protein is known to play a key role in virus assembly and budding late in infection, our data suggests that disrupting the interaction between the M and L proteins could provide a novel target for antiviral development.

## 1. Introduction

Respiratory syncytial virus (RSV) is a leading cause of severe respiratory illness globally, with infants under 5 years of age and the elderly (>65 years of age) being most impacted [1,2,3,4]. The recent development of vaccines for adults and long acting monoclonal antibody for infants (guidelines vary with country) [5], has been a major development that is forecast to have a significant positive impact in the high-income countries where they have been deployed [6]. Unfortunately, the main medical impact of RSV illness is felt in low- and middle-income countries, which mostly cannot afford the new RSV prevention measures [7]. There are no antivirals currently available to treat RSV [8] in the general population and primary treatment options are limited to supportive care [9].

RSV is an enveloped, non-segmented negative-strand RNA virus. The ~15 kb RNA genome codes for eight structural proteins (F fusion glycoprotein, G attachment glycoprotein, SH small hydrophobic protein, M matrix protein, M2-1 transcription processivity factor, N nucleocapsid protein, P phosphoprotein, and L large polymerase), two nonstructural proteins (NS1, NS2), and the M2-2 regulatory factor. The polymerase complex is responsible for both transcription and replication, includes L and P proteins, and is guided by the N protein that binds directly to RNA.

Viral polymerase complexes are central to virus replication and are hence a major target for the development of antivirals, along with the virus surface proteins that are key to infection [10]. PC751 and PC786 are two benzothienoazepine derivatives that have potent anti-RSV effect in vitro and in vivo [11,12]. PC786 is a nebulised non-nucleoside RSV polymerase (L protein) inhibitor with demonstrated antiviral effects in a human challenge study and in cell culture [11,13,14]. PC751 is a fluorinated derivative effective at suppressing RSV replication when administered intranasally in a rodent model of RSV infection [12].

To understand the molecular mechanism that underlies its antiviral effects and to confirm that it indeed targets the L protein, we undertook an escape mutant analysis using sequential passaging of the virus in the presence of increasing concentrations of PC786. As expected, we found that PC786 induced a mutation in the L gene. Interestingly, PC786 also induced a mutation in the M gene, suggesting an interaction between L and M proteins. This was unexpected as an interaction between the polymerase and matrix proteins of negative-strand viruses had not been described in the literature.

In the current study, we use in vitro, infection, transfection systems and mini-genome analysis to show an interaction between RSV L and M proteins that does not impact L protein polymerase functions but does impact M protein localisation and may impact RSV assembly.

## 2. Materials and Methods

### 2.1. Materials

PC786 [11], PC751 (compound 4a in [12]), and AZ-27 [15] compounds were synthesised by Sygnature Discovery Ltd. (Nottingham, UK; with final purities >98%). RSV A2 Large Polymerase (L) Helper Plasmid (NR-36461), RSV A2 Matrix 2-1 (M2-1) Helper Plasmid (NR-36464), RSV A2 Nucleoprotein (N) Helper Plasmid (NR-36462), and RSV A2 Phosphoprotein (P) Helper Plasmid (NR-36463) were obtained through BEI Resources, NIAID, NIH (Manassas, VA, USA). A plasmid with RSV trailer/promoter-conjugated Luciferase gene [16], mutant L protein expression vector and wild-type M protein expression vector were obtained from Creative Biogene (Shirley, NY, USA). The full-length wild-type GFP-M expression vector for mammalian expression and the 6xHis-M expression vector for bacterial expression have been described previously [17,18].

### 2.2. Cells and Virus

Human larynx epithelial (HEp-2) cells (ATCC^®^ CCL-23™), African green monkey kidney epithelial (Vero) cells (ATCC^®^ CCL-81™), and fibroblast-like (COS-7) cells (CCL-1651™) were purchased from the American Type Culture Collection (ATCC, Manassas, VA, USA) and maintained in 10% foetal bovine serum (FBS) supplemented DMEM with phenol red (# 4190-094: Life Technologies Ltd., Paisley, UK) at 37 °C, 5% CO_2_. The SV-40 immortalised human bronchial epithelial cell line BEAS-2B (ATCC) was maintained in LHC-8 medium (Invitrogen, Paisley, UK) at 37 °C, 5% CO_2_. MucilAir™ cells were provided as 24-well plate sized inserts by Epithelix Sàrl (Geneva, Switzerland). Twice weekly, MucilAir™ inserts were transferred to a new 24-well plate containing 780 µL of MucilAir™ culture medium (EP04MM), and once weekly, the apical surface was washed once with 400 µL PBS. MucilAir™ cultures were incubated at 37 °C, 5% CO_2_. RSV A2 Strain (NCPV, Public Health England, Wiltshire, UK) and RSVB Washington/18537/1962 (VR1580: ATCC) were propagated in HEp-2 cells. RNA polymerase was also purchased from ATCC and used as a donor of T7 polymerase.

### 2.3. Cytopathic Effects (CPE) Assay and Cell Viability in HEp-2 Cells

RSVA2 and RSV B Washington (WST) strains were assayed in a 96-well format resazurin-based CPE assay. Approximately 24 h after the cells were seeded into 96-well black plates (200 μL of 5% FBS phenol red-free DMEM/well) at a density of 3 × 10^4^ cells/mL, the cells were infected with RSV A2 (0.2 MOI) and RSVB WST (0.2 MOI). Plain medium was also added to non-treatment wells as non-infection controls. Immediately after the infection, the compounds and neat DMSO (0.5 µL/well) were added as appropriate to give a final concentration of 0.5% DMSO across all wells. The plates were incubated for 5 days for RSVA and clinical RSVB strains and 6 days for RSVB WST strain (37 °C, 5% CO_2_). After removing the supernatant, 200 µL of resazurin solution (0.0015% in PBS) was added to each well and the plates were incubated for a further 1 h at 37 °C/5% CO_2_. The fluorescence of each well [545 nm (excitation)/590 nm (emission)] was then determined using a multi-scanner (CLARIOstar^®^: BMG Labtech, Aylesbury Bucks, UK). The percentage inhibition for each well was calculated against infection control and the IC_50_ and IC_90_ values were calculated from the concentration–response curve generated for each test compound. For assessment of cell viability, test compounds or neat DMSO as vehicle (1 µL) were added to each well of confluent HEp-2 culture in 96-well plates (200 µL of 2.5% FBS DMEM/well) and incubated for 5 or 6 days (37 °C, 5% CO_2_). Cells were then incubated with resazurin solution and the fluorescence level of resorufin (metabolised materials) was determined as described above. Where appropriate, a 50% cytotoxicity concentration (CC_50_) value was calculated from the concentration–response curve generated for each test compound.

### 2.4. F Protein Enzyme-Linked Immunosorbent Assay (ELISA)

Approximately 72 h after BEAS-2B cells were seeded in 96-well plates (at 4 × 10^4^ cells/mL, 100 µL/well), the media in the plates was removed and replaced with 200 µL of fresh LHC-8 media. In a washout arm, compounds and DMSO as vehicle were added to the appropriate wells (1 µL/well) to give a final concentration of 0.5% DMSO across the plates. The plates were incubated at 37 °C, 5% CO_2_ for 2 h, the media was removed and replaced with 100 µL of fresh LHC-8 media and subsequently incubated at 37 °C, 5% CO_2_ for approximately 24 h. The cells were then infected with RSV A2 at an MOI of 0.01 in LHC-8 medium (100 µL/well) and incubated for 3 days (37 °C, 5% CO_2_). For non-washout plates, the compounds and DMSO (1 µL/well) were applied immediately after infection. On day 3 post-infection, supernatant was aspirated and the cells were fixed with 4% formaldehyde (100 µL in PBS solution) for 20 min at room temperature (RT), washed three times with washing buffer (WB) (200 µL: PBS containing 0.05% Tween-20), and incubated with blocking buffer (100 µL: 5% BSA in wash buffer) for 1 h. Cells were then washed with washing buffer (WB) three times (200 µL) and incubated overnight at 4 °C with anti-RSV F-fusion protein antibody [2F7] (50 µL/well: 1:2000 dilution, ab43812, Abcam plc, Cambridge, UK). After washing, cells were incubated with an HRP-conjugated anti-mouse IgG antibody (50 µL/well: 1:2000, Abcam) for 1 h. Tetramethylbenzidine (TMB) substrate (50 µL) was then added after washing twice with WB and once with PBS, and the reaction was stopped by the addition of aqueous sulphuric acid (2N; 50 µL/well). The resultant signal was determined colorimetrically (OD at 450 nm with a reference wavelength of 655 nm) in a microplate reader (Multiskan FC^®^, ThermoFisher Scientific, Abingdon, UK). Cells were then washed, and 0.5% crystal violet solution (50 µL/mL) was applied for 30 min. After washing with PBS (200 µL) twice, 1% SDS (100 µL) was added to each well, and the plates were shaken lightly for 1 h prior to reading the absorbance at 595 nm as an indicator of cell number in each well. The measured F-protein signals (OD_450–655_ readings) were corrected for cell number (OD_595_ readings) by dividing the OD_450–655_ by the OD_595_ readings.

### 2.5. Virus Infection on Air Liquid Interface (ALI) Cultured Bronchial Epithelial Cells and Plaque Assay

Infection of ALI-cultured bronchial epithelial cells and the plaque assay were performed essentially as previously described [19]. On the day of infection (‘Day 0’), the apical surface of each insert was washed once with 300 µL of sterile PBS and the inserts were then transferred to new 24-well plates containing 780 µL of fresh MucilAir™ culture medium (Epithelix Sarl, Plan-les-Ouates, Switzerland). RSV A2 was diluted in MucilAir™ culture medium to give a final inoculation concentration of 2000 PFU in 50 µL (an approximate MOI of 0.01) and incubated onto cells for one hour at 37 °C, 5% CO_2_. Virus inoculum was removed, and the inserts were washed twice with 300 µL of sterile PBS. A Day 0 sample was harvested by adding 300 µL of sterile PBS to the apical surface of each well for 5 min. The 300 µL sample was then removed and transferred to 0.5 mL tubes containing 100 µL of 50% sucrose dissolved in PBS, and the tubes were stored at −80 °C. This harvesting procedure was repeated daily until Day 7. PC786 was dosed apically onto MucilAir™ inserts. The PC786 solution diluted with PBS at 1:200 (50 µL, 0.5% DMSO in final) was applied to the apical surface and incubated at 37 °C, 5% CO_2_ for one hour before being removed. The dosing regimen was performed daily from Day 0 to Day 6 following each sample collection. On Day 5, the basal media were removed from every well and replenished with fresh MucilAir™ culture media as a necessary maintenance step for ALI-cultured bronchial epithelial cells. Virus titre in the apical wash samples was determined by plaque assay. HEp2 cells were grown in 24-well plates (Corning, Corning, NY, USA) for 48 h prior to infection in 10% FBS DMEM until they attained 100% confluency. Collected samples (above) were thawed at RT and ten-fold serial dilutions were prepared in serum-free DMEM. The growth medium from HEp2 cells was aspirated and replaced with 300 µL of serially diluted virus collections and left to infect at 37 °C, 5% CO_2_ for 4 h. The infectious media were aspirated and replaced with 500 µL plaque assay overlay (1% methylcellulose in MEM, 2% FCS) and left for 7 days at 37 °C, 5% CO_2_. Cells were fixed with ice-cold methanol for 10 min before the methanol was removed, and the cells washed twice with sterile PBS. Anti-RSV F-protein antibody [2F7] (Abcam) was diluted to a 1:150 concentration in blocking buffer (5% powdered milk (Marvel, Premier Food, Hertfordshire, UK) in 0.05% PBS-tween20) and 150 µL added to cells for 2 h at RT with shaking. Cells were washed twice with PBS before 150 µL of secondary antibody (goat anti-mouse/HRP conjugate (Dako(UK) Ltd., Ely, UK, P044701-2)), diluted 1:400 in blocking buffer, was added to cells for one hour at RT, with shaking. The secondary antibody solution was removed, and the cells were washed twice with PBS before the metal-enhanced development substrate DAB was prepared in ultra-pure water (according to manufacturer’s instructions). Each well received 150 µL of development substrate (sigmaFAST D0426, Sigma-Aldrich (Merck Life Science), Dorset, UK) until plaques were visible. Plaques were counted by eye and confirmed using light microscopy.

### 2.6. Mini-Genome-Analysis

The RSV trailer/promoter-conjugated Luciferase plasmid reported previously [16] was manufactured by Creative Biogene (Shirley, NY, USA). Briefly, four overlapping oligonucleotides were annealed to form a 238-base pair (bp) DNA fragment containing a terminal BamHI site, the upstream 32-nucleotide (nt) nonstructural protein 1 (NS1) nontranslated region, the 10 nt RSV NS1 gene start signal, a 44 nt RSV leader sequence, a 94 bp hammerhead ribozyme, a 47 bp T7 terminator, and a NotI compatible end. A 191 bp DNA fragment was synthesised in vitro*,* containing a terminal HindIII site, a 155 nt RSV trailer sequence, the 12 nt RSV L gene end sequence, a 12 nt non-translated region of RSV L, and an XhoI site (Integrated DNA Technologies, Skokie, IL, USA). These two fragments were ligated, along with a BamHI/XhoI fragment of Firefly luciferase cDNA (pGEM-luc, Promega Corp., Fitchburg, WI, USA), into the NotI and HindIII sites of pcDNA3.1, such that an antisense copy of luciferase flanked by RSV leader and trailer regulatory elements was produced by T7 polymerase transcription. On the day of transfection, growth media were removed from HEp2 cells and replaced with 50 µL of serum-free DMEM containing Vaccinia virus (expressing T7 polymerase) at an MOI of 0.1, with one row of cells receiving serum-free media only as uninfected controls. Cells were incubated at 37 °C, 5% CO_2_ for one hour, during which time plasmids were prepared for transfection using Lipofectamine 2000, as per the manufacturer’s instructions. A mastermix was prepared allowing for 0.00625 µg of M2-1-, N-, P- and Luc-encoding plasmids and 0.003215 µg of L-encoding plasmid per 60 µL of Opti-MEM to be administered per well, with a final Lipofectamine 2000 (µL) to DNA (µg) ratio of 3:1. Media containing Vaccinia virus were removed, and the transfection reagents were added to the cells and left for 2 h at 37 °C, 5% CO_2_ before a further 140 µL DMEM containing 2% FBS was added to the cells. Compounds were administered in 1 µL volumes at this time point, and the cells were further incubated for 48 h at 37 °C, 5% CO_2_. Luminescence was then detected using the Luciferase Assay System (Promega Corp.) as follows: Cells were lysed in 100 µL Cell Culture Lysis Reagent (Promega Corp.) and 20 µL from each well was transferred to a black flat-bottomed 96-well plate (Thermo Fisher Scientific, Waltham, MA, USA). Each well was injected with 100 µL Luciferase Assay Reagent and luminescence was detected for 5 s by a multi scanner (CLARIOstar^®^). A single nucleotide change, T13388C (full genome numbering), was engineered into the L-protein sequence to generate the Y1631H mutation in the L gene (Creative Biogene), and a pcDNA 3.1 (+) vector-harboured mutated L protein was also used in the mini-genome analysis.

### 2.7. Mutation Induction

HEp-2 cells, maintained in DMEM with phenol red supplemented with 10% FBS at 37 °C, 5% CO_2_, were sub-cultured twice a week. Approximately 72 h prior to beginning the experiment, cells were seeded in T75 flasks in 10% FBS DMEM to be fully confluent on the day of infection. RSV A2 (MOI of 0.2) was added to T75 flasks of HEp-2 cells, and incubated for 2 h at 37 °C, 5%CO_2_, after which the virus was removed and replaced with 12 mL of the media containing the appropriate compound dilutions. The flasks were incubated for 4–8 days at 37 °C, 5% CO_2_ until CPE was observed. The media containing floating cells and cell debris (due to CPE) were collected and centrifuged at 1200 rpm for 10 min. Cell-free supernatant was collected into sucrose solution (final sucrose concentration of 12.5% *w*/*v*) and stored at −80 °C. For the next passage, the virus stock from the previous passage (1 mL) was applied to a T75 flask containing confluent HEp-2 cells. This procedure was then repeated. The viral titre of all virus samples was determined by plaque assay. Collected samples were thawed at RT and ten-fold serial dilutions were prepared in serum-free DMEM. The growth media (10% FBS DMEM) from HEp-2 cells grown in 24-well plates was aspirated and replaced with 300 µL of serially diluted virus collections and left to infect at 37 °C, 5% CO_2_ for 4 h. The infectious media was aspirated and replaced with 1 mL of plaque assay overlay (0.3% Avicel RC-591 (FMC Biopolymer UK, Girvan, UK) in MEM, supplemented with a final concentration of 2% FBS), and left for 7 days at 37 °C, 5% CO_2_. Cells were fixed with ice-cold methanol for 10 min and then subjected to staining with 200 µL 0.1% crystal violet solution in distilled water for 1 h. The crystal violet solution was removed, and the cells were rinsed with water before plaques were counted and virus titre enumerated.

### 2.8. Virus Full Genome Sequence

Viral RNA was extracted from serially passaged virus stocks using a QIAamp Viral RNA Mini Kit (QIAGEN, Hilden, Germany) according to the manufacturer’s instructions. Approximately 55 µL of extracted RNA was then dispatched to GENEWIZ Inc. (South Plainfield, NJ, USA) for full genome sequencing using MiSeq, and single nucleotide polymorphisms (SNPs) were identified against a template RSV A2 sequence (GenBank ID: M74568.1) [11]. SNPs that appeared with a frequency of over 20% in a routinely used laboratory strain of RSV were identified as drift mutations that emerged in-house during routine passage. These nucleotide changes were used to amend the submitted RSV A2 sequence template so that a direct comparison could be made against in-house RSV A2 isolates as opposed to the published sequence mentioned above. SNPs which appeared with a frequency of over 10%, or that matched previously reported mutation sites, were analysed to see if they elicited a codon change. Silent mutations were excluded from the report.

### 2.9. PCR Genotype Assay

To confirm the genotype of the compound-treated viruses, an in house Taqman^®^ genotype assay was designed based on the sequences supplied by GeneWiz. Viral RNA was extracted from the appropriate virus samples by MagMax express and used to produce cDNA by reverse transcription using Invitrogen SuperScript VILO MasterMix (SuperScript) (Cat # 11755-250, Life Technologies), as per the manufacturer’s instructions. A mixture of 4 µL SuperScript was added to 16 µL of DEPC-treated water (Cat # 46-2224, Life technologies) per reaction and 5 µL of appropriate viral RNA solution to give a final volume of 25 µL. The RNA and SuperScript solution were centrifuged briefly at 1200 RPM to ensure the complete mixing of the solutions, and then reverse transcription was completed by incubating the samples for 10 min at 25 °C, followed by 60 min at 42 °C, and terminating the reaction at 85 °C for five minutes.

TaqMan^®^ SNP genotyping assays were designed by Life Technologies (Cat # 1500910) based on the gene sequences provided [11]. The TaqMan^®^ SNP genotyping assay solution was diluted to produce a 20× solution and per reaction 1.25 µL was added to 12.5 µL of the Taqman genotyping mastermix (Cat # 4371355, Life Technologies) and 6.25 µL of DEPC-treated water. This solution was added to appropriate wells, 20 µL per well, of a 96-well PCR plate (MicroAmp^®^ fast 96-well reaction plate, 0.1 mL, Cat # 4346907, Life Technologies) and 5 µL of the appropriate cDNA sample was added. The PCR was run using the StepOne Plus PCR machine, with enzyme activation at 95 °C for 10 min, followed by 40 cycles of 15 s at 95 °C, and 60 s at 60 °C with fluorescence measured. The data were analysed using StepOne V2.3 to determine which allele of the target gene was present in each sample.

### 2.10. Bacterial Expression of L Peptides and M Protein

The bacterial expression and purification of M protein fused to an N-terminal polyhistidine tag has been described previously [18].

The RSV L protein is large (composed of 6578 base pairs) and, for ease of handling, was expressed as truncated peptides coded by ~1000 nucleotides. Seven pairs of forward and reverse primers were designed to amplify the RSV L protein into seven fragments, with flanking BamH1 and Not1 restriction sites (primer sequences provided in Supplementary Table S1). Digested, purified inserts were ligated (T4 DNA ligase, New England Biolabs, Melbourne, Australia) with similarly digested, purified, linear pET30a vector. Ligation products were transformed into electro-competent DH10B *E. coli* at 1.8 V at 25 μF, followed by recovery in SOC medium and overnight culture in the presence of Kanamycin. Single colonies were grown in the presence of Kanamycin, and the plasmid DNA was extracted. Plasmids with L-gene inserts were identified by restriction digestion and confirmed by Sanger sequencing (Australian Genome Reference Facility, Brisbane, Australia). Confirmed plasmids for each L-gene fragment were transformed into BL21 (DE3) competent *E. coli* cells. Single colonies were picked and grown in liquid culture prior to expression in the presence of 1 mM β-D-1-thiogalactopyranoside (IPTG) for 3 h. Cells were collected by centrifugation and stored at −20 °C until required. Cells were lysed in B-PER bacterial protein extraction reagent (Thermo Fisher Scientific, Melbourne, Australia) and separated into the insoluble (IF) and soluble (SF) fractions. Samples were analysed by SDS-PAGE, followed by Western blotting and immunoprobing with primary anti-poly histidine (Roche, Basel, Switzerland) and secondary HRP anti-mouse antibody. Bound proteins were visualised by enhanced chemiluminescence (ECL, Western Lighting, Perkin Elmer, Melbourne, Australia) using the LiCor Odyssey imaging system [20].

### 2.11. Protein–Protein Overlay Binding Assay

L peptide samples (IF or SF) were separated by SDS-PAGE, followed by Western blotting. The membrane was blocked with bovine serum albumin (BSA) before incubation in purified M protein overnight at 4 °C with shaking. The blot was washed thoroughly, and bound M protein was detected by mouse anti-M primary antibody (Mab B50) [21], followed by anti-mouse HRP-conjugated secondary antibody and ECL as above.

### 2.12. Mammalian Expression of L Peptides and M Protein

The mammalian expression construct encoding full-length wild-type GFP-M has been described previously [22]. A mutant construct was also generated, where the Valine at 153 was mutated to Alanine (Mmut, M-V153A) using site-directed mutagenesis, followed by recombination into the pEpiDESTC vector [22].

Sequences for L fragments 5, 6, and 7 were amplified by PCR from the full-length RSV genome, restriction-digested, and inserted into the mCherry-C1 plasmid vector to encode mCherry-L5, -L6, -L7. A mutant L6 construct was also generated, where the Tyrosine at position 1631 was mutated to Histidine (L6mut, L6Y1631H).

Overnight subconfluent monolayers of COS-7 cells cultured on glass coverslips (Proscitech, Brisbane, Australia) were transfected with plasmid DNA using Lipofectamine 2000 (Invitrogen) as recommended by the manufacturer. Then, pEPI-DESTC plasmids encoding GFP-M or GFP-Mmut were co-transfected into COS-7 cells with mCherry-C1 plasmids encoding mCherry-L5, -L6, -L6mut, or -L7. Control samples were co-transfected with pEPI-DESTC and mCherry-C1. In some instances, cells were treated with PC751 or PC786 for 12 h before imaging. At 18 h post-transfection, cells were incubated for 10 min with Hoechst 33342 and imaged live (see below, Section 2.13).

### 2.13. Confocal Microscopy

*Transfection.* At 18 h post-transfection, cells were imaged live, as previously, using a Nikon Ti Eclipse confocal laser-scanning microscope (CLSM) with a Nikon 60×/1.40 oil immersion lens (Plan Apo VC OFN25 DIC N2; optical section of 0.5 μm) and the NIS Elements AR software, (version 4.0, Nikon, Tokyo, Japan); data from four individual scans were averaged to obtain the final images for each channel (GFP, mCherry, and Hoechst).

*Infection.* HEp-2 cells were infected with wild-type (A2) RSV or the induced mutant (A2-751, A2-786) at MOI = 1 and processed for immunofluorescence at the indicated times. In some instances, cells were treated with the reversible nuclear export inhibitor, Verdinexor (KPT335), for 12 h from 18 h post-infection, before the immunofluorescence assay. Cells were fixed with paraformaldehyde (4% in PBS) and membranes permeabilised with Triton X100 (0.5% in PBS). Cells were incubated with goat polyclonal antibody to RSV with rabbit polyclonal antibody to F protein or mouse monoclonal to M protein, followed by fluorophore-conjugated secondary antibodies. Cells were incubated for 10 min in Hoechst 33342, before mounting the coverslips on microscope slides using DAKO antifade mounting medium. Cells were imaged using a Nikon Ti Eclipse CLSM, as above.

### 2.14. Image Analysis

Where indicated, digital images obtained by CLSM were analysed as described previously, using ImageJ v1.53 software, to determine the fluorescence intensity above background (Fb) in the nucleus (Fn) compared to that in the cytoplasm (Fc), and to determine the nuclear to cytoplasmic fluorescence ratio (Fn/c). The co-localisation of mCherry-L peptides with GFP-M or GFP-Mmut was analysed using the Coloc2 plugin within ImageJ. Pearson’s correlation coefficient was used to determine the overlap between two images by measuring their pixel-by-pixel (red and green) signal covariance.

### 2.15. Statistical Analysis

Results are represented as the mean ± standard error of the mean. The IC_50_ and CC_50_ values were calculated with Dotmatics software (v7.1, Dotmatics Ltd., Hertfordshire, UK) or GraphPad Prism 6^©^ (GraphPad Software Inc., Boston, MA, USA). The safety index was calculated as the ratio of the CC_50_ and IC_50_ values. Multiple comparison was performed by ANOVA followed by Dunnett’s multiple comparison test. In some cases, non-parametric Kruskal–Wallis analysis followed by Dunn’s multiple comparison test was conducted. The comparison between two groups was performed by unpaired *t*-test with Welch’s correction or Mann–Whitney test. Statistical significance was defined as *p* < 0.05.

## 3. Results

### 3.1. In Vitro Anti-RSV Profile of Compounds

The antiviral activities of PC786 and PC751 were assessed in CPE assays in vitro*,* using HEp-2 and laboratory-adapted RSV strains (RSV A2, RSV B Washington (WST)). Both PC786 and PC751 potently inhibited CPE induced by RSV A2 and RSVB WST (Table 1). These inhibitory values were much more potent than the known RSV L-protein inhibitor, AZ-27. PC751, specifically, was equally potent against RSV A and RSV B. Neither PC786 nor PC751 affected cell viability at concentrations up to 14 µM and consequently showed a large safety margin with respect to mammalian cell toxicity (Table 1). Both PC786 and PC751 produced a potent, concentration-dependent inhibition of the expression of luciferase, driven by a mini-genome construct comprising the L, M2-1, N and P proteins of RSV A2 in HEp-2 cells. Both also inhibited F-protein expression post RSV A2 infection in the bronchial epithelial cells, BEAS-2B. The persistence of action of PC786 and PC751 was assessed by RSV F-protein cell-based ELISA on RSV A2-infected BEAS-2B cells. The procedure relies upon determining the effect of a 24 h washout period on the potency of PC786/PC751 compared with the compound’s activity when treated just after virus inoculation and left in situ, i.e., omitting a wash-out. As shown in Table 1, after a 24 h washout, the activity persisted (only 2.7–6.1-fold reduction of IC_50_), indicating that short exposure to PC786/751 has a sustained antiviral effect compared to AZ-27.

### 3.2. Antiviral Effects on RSV A2-Infected Air-Liquid Interface (ALI)-Cultured Human Bronchial Epithelium

**Figure 1:**
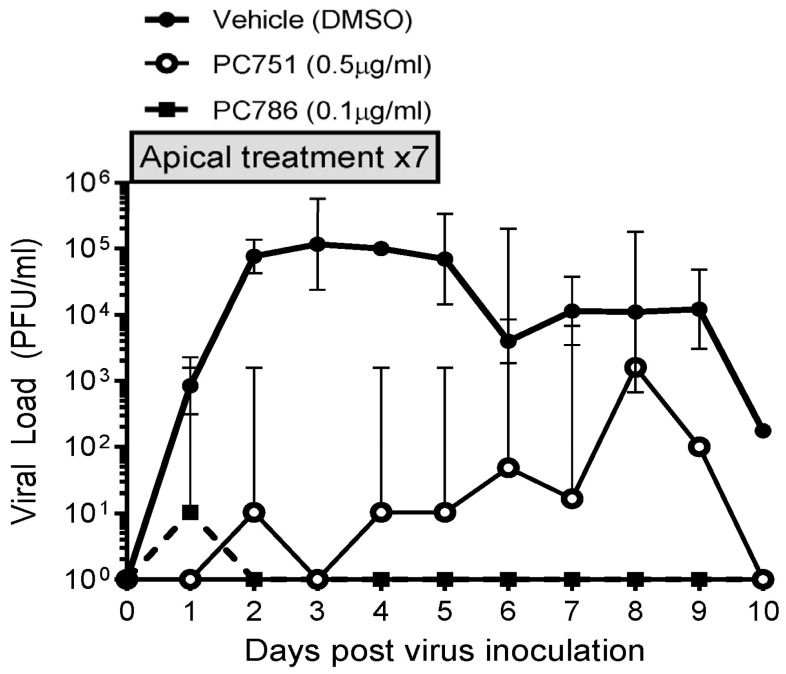
Changes in viral load in vehicle, PC786- and PC751-treated ALI-cultured bronchial epithelium post RSV (0.002 MOI) apical inoculation. PC786 or PC751 was added to the apical surface, once daily from Day 0 (1 h after virus inoculation) to Day 6. The assay was conducted in triplicate, and the graph indicates geometric mean with 95% CI.

**Figure 2:**
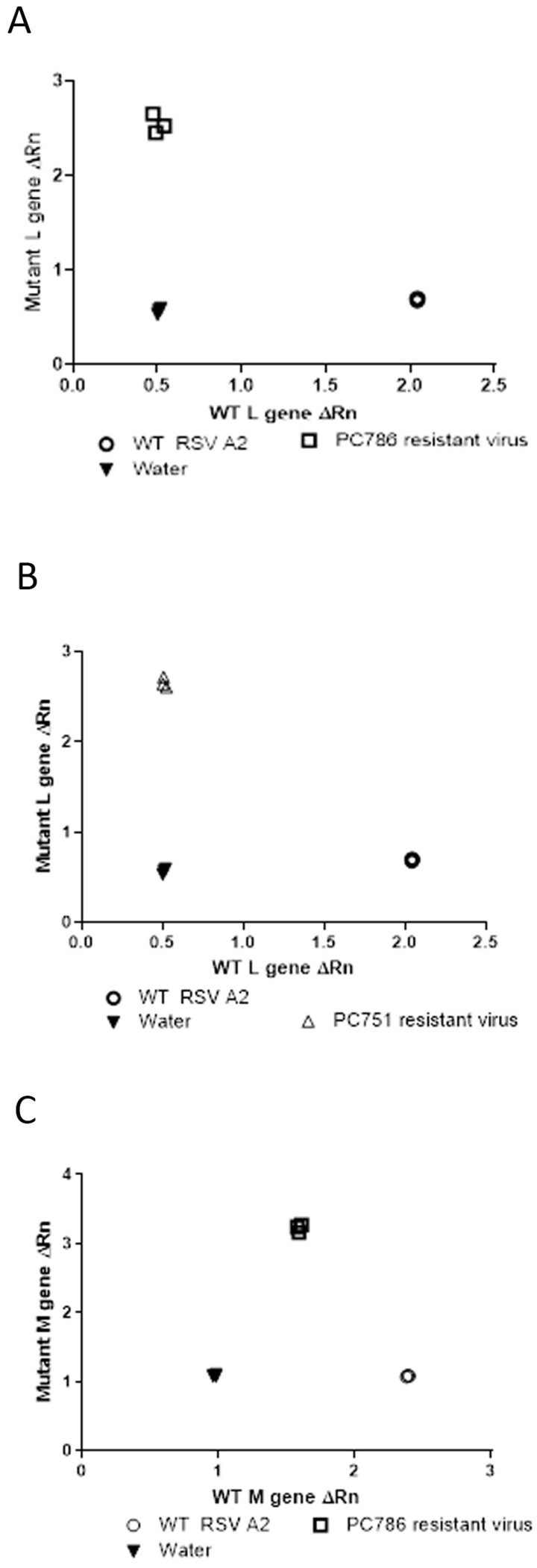
SNP genotype analysis of the L-gene of RSV A2 treated with PC786 (**A**), PC751 (**B**) or wild-type RSV A2 (passage 6), and also of the M-gene of RSV A2 treated with PC786 (**C**). The axes show the relative fluorescence of the probes specific to either the WT or mutant sequence of the L-gene or M-gene.

The antiviral effects of PC786 and PC751 were evaluated using ALI-cultured fully differentiated human primary bronchial epithelium. These cells undergo extensive mucociliary differentiation, resulting in cultures with morphological characteristics similar to those observed in the normal human respiratory epithelium. Following inoculation with a low level of RSV A2 (0.002 MOI), the quantity of RSV in apical washes, as determined by plaque assay, in the absence of any antiviral compounds increased from Day 1 to a peak at Day 2–3, and then waned gradually and modestly up to Day 10 (Figure 1). Treatment with PC786 to the apical surface, once daily from Day 0 (1 h after virus inoculation) to Day 6, induced a marked inhibition of RSV A2 replication. This effect commenced immediately (observable within 24 h) following PC786 treatment on Day 0 post-inoculation, and reduced viral quantity to below detectable limits, for the duration of the experiment, at a dose of 0.1 µg/mL. On the other hand, PC751 treatment (0.5 µg/mL) completely reduced viral load initially on Day 1, but it increased over time to be equal to no treatment at Day 8 post-inoculation, although we did not find any mutation of RSV A2 during this rebound (Figure 2).

### 3.3. Analysis of Escaped Mutant

RSV A2 viruses were repeatedly passaged in HEp-2 cells in the presence of increasing concentrations of PC786 or PC751 (from 1.4 nM to 4366 nM; up to 8700-fold higher than IC_50_ value). After the 6th passage, PC786 escape mutants were obtained, which exhibited IC_50_ values in the CPE assays that were >650-fold weaker compared with the control virus, and PC751 escape mutants that were >91,000-fold weaker compared with the control virus. When the complete RSV genome was sequenced, the mutant at the 6th passage showed two amino acid substitutions had occurred with high frequency (99%); the first being tyrosine 1631 to histidine or leucine (Y1631H, Y1631L) in the RSV L-gene in both PC786 and PC751-treated cells. In addition, the exchange of valine 153 to alanine or methionine (V153A or V153M) in the RSV M-gene was seen in PC786-treated cells only (99% frequency). Both mutants arose in the 4th passage, but neither were confirmed in the 2nd passage. Phenotypic validation of the Y1631 mutation was conducted using the RSV mini-genome assay, in which the plasmid encoding the Y1631H-mutated RSV L-gene together with wild-type N, P, and M2-1 plasmids were co-transfected into HEp-2 cells [11]. It was found that PC786 showed a >187-fold shift in the resulting IC_50_ value obtained from the mini-genome assay when run against the mutated L-protein (IC_50_: 0.48 nM in wild-type L protein, 89.7 nM in mutant L protein).

### 3.4. PCR SNP Analysis

Genotyping analysis of the escaped mutant of the compound-treated viruses was conducted by SNP PCR analysis using the genetic sequences provided by GeneWiz (only for target L protein at Y1631H, and M protein V153A) as previously reported [11]. The outcome of this analysis compared the relative fluorescence of the probes specific to the wild-type and mutant gene sequences, and these values were used to determine whether the samples were homozygous for either sequence or heterozygous. The results using the primers and probes for the known polymorphism in the L-gene showed that PC786- and PC751-resistant virus was homozygous for the mutation (Y1631H) (Figure 2A,B) and the wild-type virus was shown to be homozygous for the wild-type gene sequence. In addition, as shown in Figure 2C, the PC786-induced mutant virus showed M-protein mutation, but the PC751-induced mutant did not.

### 3.5. PC786 Directly Impacts M Protein Localisation

**Figure 3:**
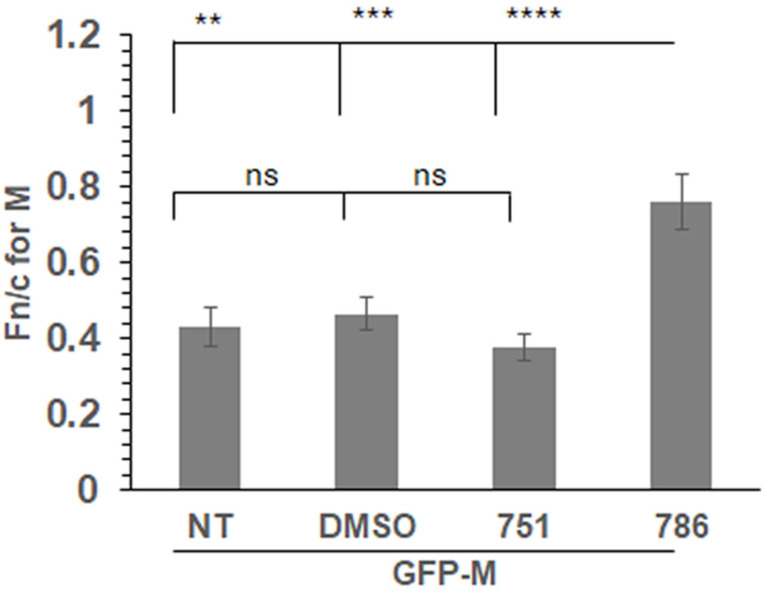
PC786 treatment results in increased nuclear retention of GFP-M. Cells transfected to express GFP-Mwt were treated with 100 nM PC751 or PC786, DMSO (vehicle), or left untreated (NT) for 12 h before imaging live at 24 h post transfection. Fn/c for M protein was calculated using the formula Fn/c = (Fn − Fb)/(Fc − Fb), where Fn = nuclear fluorescence, Fc = cytoplasmic fluorescence, Fb = background autofluorescence. Unpaired Student’s *t* test was used to determine statistical differences indicated on the graphs. Statistical significance was set at *p* < 0.05. **—*p* < 0.001, ***—*p* < 0.0001, ****—*p* < 0.00001, ns—non significant.

We expressed a GFP-tagged wild-type M protein (GFP-M) in transfected cells followed by treatment with PC751, PC786, or vehicle (DMSO) for 12 h. Localisation of GFP-M was assessed by live cell CLSM, followed by quantitative image analysis of digital confocal images. The relative localisation of M in the nucleus and the cytoplasm is denoted by Fn/c, which represents the fluorescence in the nucleus relative to the fluorescence in the cytoplasm, corrected for background fluorescence [17,23]. In the absence of any treatment (NT), or when treated with the vehicle (DMSO), GFP-M was clearly cytoplasmic (Figure 3, Fn/c~0.40). Treatment with PC786 resulted in a significant change in the localisation of GFP-M, becoming almost equally distributed in nucleus and cytoplasm (Fn/c~0.80). In contrast, there was no effect of similar treatment with PC751. These data correlate perfectly with the forced mutation analysis, wherein PC751 treatment results in a mutation in L protein only, while PC786 treatment results in mutation in the L as well as M protein.

### 3.6. PC786-Induced Mutations Affect RSV Assembly and Budding

Infectious viruses with mutations in either L alone (Y1631H; A2-751) or in both L and M (Y1631H, V153A; A2-786) were compared with wild-type RSV (A2) for the subcellular localisation of F and M proteins through the lifecycle. F and M proteins are major structural proteins and drive the infection (F), assembly (M), and budding (F, M) of RSV.

**Figure 4:**
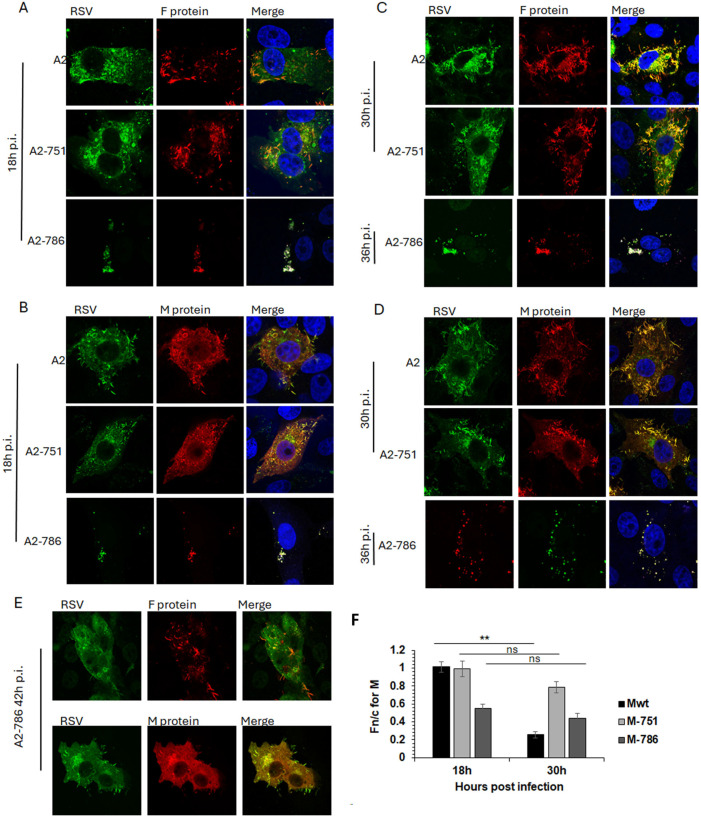
A2-786 has abnormal assembly and budding compared to wild-type A2. HEp2 cells were infected with A2 wild-type or its mutants, A2-751 (mutation in L only), A2-786 (mutation in L and M) at MOI = 1. Cells were fixed at different times, permeabilised and probed with anti-RSV antibody (recognises F, N, P, M proteins) and either anti-F protein or anti-M protein antibodies. Cells were incubated with Alexa488 (green) or Alexa547 (red) secondary antibodies, stained with Hoechst (blue nuclear stain) and mounted on slides. Slides were viewed on Nikon Ti confocal system and digital images collected as described in text. Single images representative of at least 15 images from two different experiments are shown. Colocaliation is denoted by yellow colour in the images labelled ‘merge’. (**A**,**B**) Cells fixed at 18 h post infection. (**C**,**D**) Cells fixed at 30 h (A2-751) or 36 h (A2-786) post-infection. (**E**) A2-786 infected cells fixed at 42 h post-infection. (**F**) The Fn/c for M at 18 h and 30 h post-infection was calculated (as in Figure 3) for digital images such as shown in (**A**–**D**). Unpaired Student’s *t*-test was used to determine statistical differences indicated on the graph. Statistical significance was set at *p* < 0.05. **—*p* < 0.001, ns—non significant.

In cells infected with A2, F protein was present at the cell surface at 18 h post-infection (p.i.), with clear association with filamentous RSV at 30 h p.i. (Figure 4A,C, top row), as previously described. M protein was present in characteristic cytoplasmic inclusion bodies (IBs) and in cell surface protrusions from 18 h p.i. (Figure 4B, top row), becoming associated with filamentous RSV at 30 h p.i. (Figure 4D, top row). A similar trend for F and M localisation was observed in cells infected with A2-751 (Figure 4 compare images in the middle row, labelled A2-751, with images in the top row, labelled A2). Of note are the clearly distinct filamentous RSV particles at 30 h p.i. in cells infected with both viruses, strongly suggesting comparable replication kinetics.

In contrast, A2-786 had a clearly delayed lifecycle, with filamentous RSV becoming visible only at 42 h p.i. (Figure 4A–D, images labelled A2-786, and E). At 18 h p.i., a minimal expression of F and M proteins was observed, which increased at 30 h p.i., forming some filamentous RSV at 42 h p.i. Interestingly, clear IBs could not be seen, although M localised to cytoplasmic puncta, resembling the previously identified cytoplasmic assembly granules observed late in infection [24].

We have previously shown that M protein localises to the nucleus of infected cells in order to inhibit host cell transcription [23,25]. Importantly, a recombinant RSV with a mutant M unable to inhibit host transcription is significantly attenuated [23]. Hence, we next investigated whether the impaired nuclear transport of M in A2-786 may underlie the observed delay in replication kinetics (Figure 4F).

The subcellular localisation of M protein in cells infected by A2, A2-751, or A2-786 was assessed by quantitative image analysis of digital confocal images probed for M, such as those shown in Figure 4A–E. In cells infected with A2, M protein was distributed equally in the nucleus and cytoplasm at 18 h p.i. (Fn/c~1.0), becoming clearly cytoplasmic (Fn/c < 1.0) at 30 h p.i., as expected (Figure 4F, black columns, labelled Mwt) [25,26]. In cells infected with A2-751, M protein was localised equally in nucleus and cytoplasm at 18 h p.i., exactly like that in A2-infected cells (compare black columns with light grey columns, labelled 18 h). At 30 h p.i., M became relatively more cytoplasmic (Fn/c < 1.0); however, this change in localisation was not significantly different from that at 18 h p.i., suggesting that the mutation in L protein is associated with impaired cytoplasmic localisation of the M protein. The M protein in A2-786-infected cells was clearly cytoplasmic (Fn/c < 1.0) at 18 h p.i. (dark grey column), suggesting that the mutation in M impairs its ability to localise to the nucleus. It remained cytoplasmic at 30 h p.i.

### 3.7. RSV L Protein Associates with M Protein In Vitro

**Figure 5:**
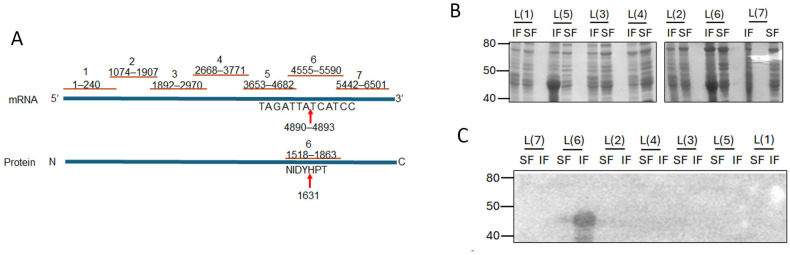
M protein binds to fragment 6 (1518 aa to 1863 aa) of L protein. (**A**) Schematic showing the position of the L-protein fragments and the mutation in fragment 6. The cDNA is shown, with L protein below. Red lines indicate the length of each fragment. Arrows point to mutation. (**B**) Each of the L-gene fragments shown in A was cloned into pET30a vector for expression in bacteria. Each clone was expressed in *E. coli* and lysate separated into soluble (SF) and insoluble (IF) fractions using B-PER reagent (Pierce). Equivalent volumes of each fraction were separated by SDS-PAGE and stained with Coomassie Brilliant Blue. Molecular weights in kDa are shown on the left and fragment identity on the top of the gel. (**C**) Protein–protein overlay binding assay. Proteins separated on SDS-PAGE as in B were transferred to a nitrocellulose membrane. The membrane was blocked and probed with purified wild-type M protein. Bound M protein was detected with mouse antibody to M protein followed by secondary antibody and development using ECL. Luminescent bands were detected on a LiCor Odyssey Fc. Molecular weights in kDa are shown on the left and fragment identity above the blot.

Given the small size of PC786 (715.83 Da) and that it impacts both L and M proteins, we hypothesised that there may be an interaction between RSV L and M proteins which brings the proteins close together, enabling PC786 to bind to both. In the first instance, we tested our hypothesis in vitro, using bacterially expressed recombinant proteins. The large size of the RSV L protein presents a challenge for recombinant expression in bacteria and was instead expressed as seven sequential fragments/peptides that had overlapping ends (Figure 5A). Each L peptide was present in both insoluble and soluble fractions of transformed BL21DE3 cells (Figure 5B). Full-length RSV 6xhis-M bound to the L peptide 6 (L(6)) in the insoluble fraction (Figure 5C), with some low-level binding observed with the soluble fraction. This is interesting, as the PC751- and PC786-induced mutation in L (Y1631H) is present within the sequence encoded by L(6) peptide (see schematic, Figure 5A).

**Figure 6:**
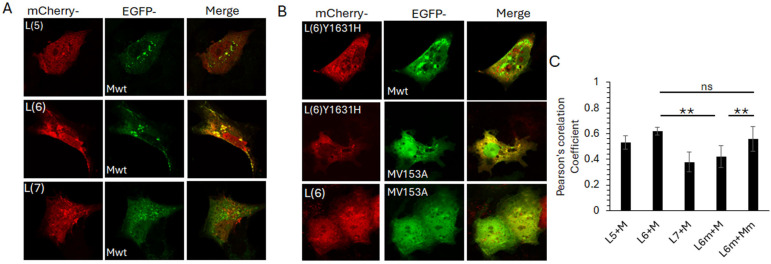
Interaction between fragment 6 of L and M protein is modulated by Y1631 and V153 in L and M proteins, respectively. (**A**) L-gene fragments 5, 6, and 7, shown in Figure 5, were cloned into pmCherry-C1, with a N-terminal mCherry (red) fusion tag. Each plasmid was co-transfected into COS-7 cells with wild-type M (Mwt) cloned in frame with an N-terminal EGFP (green) tag. Images were acquired 24 h later by live cell confocal microscopy on a Nikon Ti system using NIS Elements software. Single cells for each co-transfection are shown, representative of at least 20 cells and three independent experiments. Colocalization is indicated by yellow colour in the images labelled ‘merge’. (**B**) Site-directed mutagenesis was used to mutate fragment 6 (Y1631H) and Mwt (V153A). Mutants were cloned into pmCherry-C1 and pEGFP-DestC, respectively. Plasmids were co-transfected into COS-7 cells to express the requisite L fragment with Mwt or M (V153A), followed by live cell microscopy as for A. (**C**) Images such as those in A, B were analysed for co-localisation of red and green pixels using the Coloc2 plugin within Fiji. Mean ± SEM of Pearson’s correlation coefficient is depicted. L6m—mutated fragment 6 (Y1631H). Mm—mutated M (V153A). Statistical significance was set at *p* < 0.05. **—*p* < 0.001, ns—non significant.

To determine if the observed L6-M interaction is sustained in the cellular context, we co-expressed the selected mCherry-tagged L peptides with GFP-tagged full-length M protein, followed by live cell confocal microscopy (Figure 6). Clear co-localisation of mCherry-L6 and GFP-M was observed in perinuclear puncta, with some co-localisation also observed between mCherry-L5 and GFP-M (Figure 6A upper images, note that only some of the puncta positive for GFP-M are also positive for mCherry-L5). No co-localisation was observed between mCherry-L7 and GFP-M (Figure 6A, bottom row, note the absence of any yellow-coloured regions in the image labelled ‘merge’). All mCherry-L peptides were present in the nucleus and cytoplasm, with some localisation in perinuclear, cytoplasmic punctate structures (Figure 6A, first column, labelled mCherry). GFP-M was present mostly in the cytoplasm, with significant localisation in perinuclear, cytoplasmic punctate structures (Figure 6A, middle column, labelled EGFP).

We next investigated the impact of the observed PC751 (Y1631H)- and PC786 (Y1631H, V153A)-forced mutations on the mCherry-L6 and GFP-M interaction (Figure 6B). In contrast to its wild-type counterpart, mCherry-L6m localised primarily to the cytoplasm and cytoplasmic punctate structures (compare the top row of 6B with the first column of 6A). No co-localisation was observed between mCherry-L6m and GFP-M, with both proteins localised in separate cytoplasmic punctate structures (Figure 6B, top row, image labelled ‘merge’, note the green and red puncta and the absence of yellow-coloured puncta). In contrast to its wild-type counterpart, GFP-Mm was present in the nucleus as well as the cytoplasm, and in cytoplasmic punctate structures (compare middle row of Figure 6B with the middle column in Figure 6A). Some localisation of GFP-Mm was observed with mCherry-L6m in cytoplasmic puncta (note that some, not all, GFP-M positive puncta are also positive for mCherry-L6m, as demonstrated by the yellow colour in the image labelled ‘merge’). No clear co-localisation could be observed between mCherry-L6 and GFP-Mm (bottom row, Figure 6B).

Co-localisation analysis using the Coloc2 plugin within Fiji (ImageJ) confirmed the observations. mCherry-L6 co-localised with GFP-M (Pearsson’s coefficient, PC = 0.62 ± 0.03). There was a reduced co-localisation of mCherry-L6m with GFP-M (PC = 0.42 ± 0.5) but no change in the co-localisation of mCherry-L6m with GFP-Mm (PC = 0.63 ± 0.05). Co-localisation was also observed between mCherry-L5 and GFP-M (PC = 0.51 ± 0.06), albeit lower than that between mCherry-L6 and GFP-M.

## 4. Discussion

During a targeted anti-RSV medicinal chemistry campaign, two potent L-protein inhibitors (PC786 and PC751) were identified [11]. Both compounds were equally potent in inhibiting RSV replication in HEp-2 cells and monolayer bronchial epithelial cell line BEAS-2B, as well as inhibition of polymerase activity in minigenome analysis. Both compounds were also confirmed to have persistent effects on BEAS-2B cells. However, PC786 was more potent than PC751 on ALI-cultured primary human bronchial epithelium that closely replicates the human airway wall. Virus rebound was observed during PC751, but not PC786—treatment without induction of mutation—suggesting that PC786 has additional anti-RSV mechanisms compared to PC751. The rebound was not due to the poor duration of action of PC751 as we proved both compounds have a long cell residency, using BEAS-2B cells. Repeated treatment with escalating concentrations of RSV A2-infected HEp-2 cells revealed both inhibitors led to a Y1631H mutation in the L protein, but only PC786 induced a mutation in the M protein (V153A). By L-protein fragment and M-protein binding analysis, we showed that the M protein interacts with the 1392–1735 amino acid region of the L protein, where PC786 potentially binds.

In addition, the PC786 treatment of RSV-infected cells or infection with PC786-induced mutant RSV was found to increase M protein nuclear localisation, concomitant with delayed fusion protein localisation at the budding viral filaments. These data support our previous finding that the efficient cytoplasmic localisation of M protein is required for optimal RSV assembly [22]. The data from the current study suggest that a direct interaction between L and M proteins has at least some effect on M’s cytoplasmic retention. As M protein is known to play a key role in virus assembly and budding late in infection [22,27], our data suggests that disrupting the interaction between M and L could provide a novel target for antiviral development.

The L–M interaction identified in this study is intriguing, as previous work has shown that M associates with viral ribonucleocapsid complexes (vRNP) in IBs to inhibit transcriptase activity [26], raising the possibility that M can directly influence L’s activity [24,28,29]. Electron microscopy of purified and cell-associated RSV suggests that M is present in close proximity to RNA and that its presence influences the organisation of RNP [29], providing some evidence for the possibility that M influences L’s activity.

Although the N–RNA, N–P, and P–L interactions have been resolved individually, the 3D structure of the full vRNP is not known, making it difficult to predict the location of M. Limited 3D structures of the RSV vRNP have found that the genomic RNA is present in a surface groove around the N-protein core [30,31]. The association of the L-P polymerase with this structure has been hypothesised [31] but not yet resolved structurally. Understanding the role of the L–M interaction in RSV infection awaits further structural understanding of the M–vRNP interactions.

High-resolution microscopy images from cells infected with a recombinant RSV with a flag-tagged L protein show that L protein is present throughout the IBs [24,29], presumably, along with the P protein. The location of N, M, M2-1 in IBs is not yet known; however, antibody-based investigations suggest M is present on the outside of IBs. If true, this would suggest that only a sub-population of L protein is accessible for interaction with M, which argues against a direct effect of M on L’s activity.

Given our previous study showing that M protein interacts with microfilaments in RSV-infected cells [18] and that vRNPs associate with microfilaments only in the presence of M [32,33], could the L–M interaction on the periphery of the IBs facilitate the movement of the vRNPs to areas of RSV assembly?

There are some limitations to this study. First, as the 3D structure of the full L protein has not been resolved, we are unable to show the in silico docking model of PC786, especially at the binding site between L and M proteins. Second, M–L protein binding was only demonstrated in submerged cell culture, and future studies should be conducted in primary bronchial epithelium. The immunoprecipitation of the L and M proteins can be the way to prove the association of these two proteins only if specific antibodies against L and M proteins are available. Third, if the M protein mutant virus (V153A) is produced by genome engineering, it would be a powerful tool to analyse the role of M protein further. Future work in this collaboration is aimed at unravelling some of the questions raised by this study.

## 5. Conclusions

We have described a novel association of L and M proteins of RSV, and targeting the association is a promising approach in combatting RSV infection.

## 6. Patents

US10626126B1 Pulmocide Limited.

US9732098B2 Pulmocide Limited.

## Figures and Tables

**Table 1:** In vitro anti-RSV activity.

	RSV A	RSV B	B/A	Cytotoxicity	Minigenome	F-Protein [BEAS2B]	
	IC_50_ (nM)	IC_50_ (nM)	Ratio IC_50_	CC_50_ (nM)	IC_50_ (nM)	IC_50_ (nM)	Fold reduction
AZ-27	26.4	>1580	>60	>15800	10.2	3.2	35
PC786	0.50	27.3	55	>13970	0.48	0.13	6.1
PC751 *	1.4	2.1	1.5	>14310	2.02	0.26	2.7

* Compound 4a in [12].

## Data Availability

All data will be available on request.

## Supplementary Materials

The following supporting information can be downloaded at: https://www.mdpi.com/article/10.3390/v16121881/s1, Table S1. PCR primer sequences for cloning of L protein fragments.

## References

[1] Ohbayashi H., Sakurai T., Himeji D., Fukushima Y., Takahashi H., Kiyosue A., Sabater Cabrera E., Matsuki T., Molnar D., Preckler Moreno V. (2024). Burden of respiratory syncytial virus infections in older adults with acute respiratory infection in japan: An epidemiological study among outpatients. Respir. Investig..

[2] Poshtiban A., Wick M., Bangert M., Damm O. (2024). Burden of respiratory syncytial virus (rsv) infection in germany: A systematic review. BMC Infect. Dis..

[3] Bender R.G., Sirota S.B., Swetschinski L.R., Dominguez R.M.V., Novotney A., Wool E.E., Ikuta K.S., Vongpradith A., Rogowski E.L.B., Doxey M. (2024). Global, regional, and national incidence and mortality burden of non-covid-19 lower respiratory infections and aetiologies, 1990–2021: A systematic analysis from the global burden of disease study 2021. Lancet Infect. Dis..

[4] Heemskerk S., van Heuvel L., Asey T., Bangert M., Kramer R., Paget J., van Summeren J. (2024). Disease burden of rsv infections and bronchiolitis in young children (<5 years) in primary care and emergency departments: A systematic literature review. Influenza Other Respir. Viruses.

[5] DeVolld T., Rivard K.R. (2024). Rsv immunization in adults and children: A practical guide for clinicians. Cleve Clin. J. Med..

[6] Kieffer A., Beuvelet M., Moncayo G., Chetty M., Sardesai A., Musci R., Hudson R. (2024). Disease burden associated with all infants in their first rsv season in the uk: A static model of universal immunization with nirsevimab against rsv-related outcomes. Infect. Dis. Ther..

[7] MacNeil A., McMorrow M. (2024). Rsv burden and prevention in children in lmics. Lancet Glob. Health.

[8] Oti V.B., Idris A., McMillan N.A.J. (2024). Intranasal antivirals against respiratory syncytial virus: The current therapeutic development landscape. Expert. Rev. Anti Infect. Ther..

[9] RSV GOLD—ICU Network Collaborators (2024). Respiratory syncytial virus infection among children younger than 2 years admitted to a paediatric intensive care unit with extended severe acute respiratory infection in ten gavi-eligible countries: The rsv gold-icu network study. Lancet Glob. Health.

[10] Tripp R.A., Martin D.E. (2024). Antiviral agents and therapeutics against respiratory viruses. Expert Opin. Investig. Drugs.

[11] Coates M., Brookes D., Kim Y.I., Allen H., Fordyce E.A.F., Meals E.A., Colley T., Ciana C.L., Parra G.F., Sherbukhin V. (2017). Preclinical characterization of pc786, an inhaled small-molecule respiratory syncytial virus l protein polymerase inhibitor. Antimicrob. Agents Chemother..

[12] Fordyce E.A.F., Brookes D.W., Lise-Ciana C., Coates M.S., Hunt S.F., Ito K., King-Underwood J., Onions S.T., Parra G.F., Rapeport G. (2017). Discovery of novel benzothienoazepine derivatives as potent inhibitors of respiratory syncytial virus. Bioorg. Med. Chem. Lett..

[13] Brookes D.W., Coates M., Allen H., Daly L., Constant S., Huang S., Hows M., Davis A., Cass L., Ayrton J. (2018). Late therapeutic intervention with a respiratory syncytial virus l-protein polymerase inhibitor, pc786, on respiratory syncytial virus infection in human airway epithelium. Br. J. Pharmacol..

[14] DeVincenzo J., Cass L., Murray A., Woodward K., Meals E., Coates M., Daly L., Wheeler V., Mori J., Brindley C. (2022). Safety and antiviral effects of nebulized pc786 in a respiratory syncytial virus challenge study. J. Infect. Dis..

[15] Tiong-Yip C.L., Aschenbrenner L., Johnson K.D., McLaughlin R.E., Fan J., Challa S., Xiong H., Yu Q. (2014). Characterization of a respiratory syncytial virus l protein inhibitor. Antimicrob. Agents Chemother..

[16] Dochow M., Krumm S.A., Crowe J.E., Moore M.L., Plemper R.K. (2012). Independent structural domains in paramyxovirus polymerase protein. J. Biol. Chem..

[17] Ghildyal R., Ho A., Wagstaff K.M., Dias M.M., Barton C.L., Jans P., Bardin P., Jans D.A. (2005). Nuclear import of the respiratory syncytial virus matrix protein is mediated by importin beta1 independent of importin alpha. Biochemistry.

[18] Shahriari S., Wei K.J., Ghildyal R. (2018). Respiratory syncytial virus matrix (m) protein interacts with actin in vitro and in cell culture. Viruses.

[19] Ito K., Daly L., Coates M. (2023). An impact of age on respiratory syncytial virus infection in air-liquid-interface culture bronchial epithelium. Front. Med..

[20] Ghildyal R., Teng M.N., Tran K.C., Mills J., Casarotto M.G., Bardin P.G., Jans D.A. (2022). Nuclear transport of respiratory syncytial virus matrix protein is regulated by dual phosphorylation sites. Int. J. Mol. Sci..

[21] Orvell C., Norrby E., Mufson M.A. (1987). Preparation and characterization of monoclonal antibodies directed against five structural components of human respiratory syncytial virus subgroup b. J. Gen. Virol..

[22] Ghildyal R., Ho A., Dias M., Soegiyono L., Bardin P.G., Tran K.C., Teng M.N., Jans D.A. (2009). The respiratory syncytial virus matrix protein possesses a crm1-mediated nuclear export mechanism. J. Virol..

[23] Li H.M., Ghildyal R., Hu M., Tran K.C., Starrs L.M., Mills J., Teng M.N., Jans D.A. (2021). Respiratory syncytial virus matrix protein-chromatin association is key to transcriptional inhibition in infected cells. Cells.

[24] Blanchard E.L., Braun M.R., Lifland A.W., Ludeke B., Noton S.L., Vanover D., Zurla C., Fearns R., Santangelo P.J. (2020). Polymerase-tagged respiratory syncytial virus reveals a dynamic rearrangement of the ribonucleocapsid complex during infection. PLoS Pathog..

[25] Ghildyal R., Baulch-Brown C., Mills J., Meanger J. (2003). The matrix protein of human respiratory syncytial virus localises to the nucleus of infected cells and inhibits transcription. Arch. Virol..

[26] Ghildyal R., Mills J., Murray M., Vardaxis N., Meanger J. (2002). Respiratory syncytial virus matrix protein associates with nucleocapsids in infected cells. J. Gen. Virol..

[27] Bajorek M., Caly L., Tran K.C., Maertens G.N., Tripp R.A., Bacharach E., Teng M.N., Ghildyal R., Jans D.A. (2014). The thr205 phosphorylation site within respiratory syncytial virus matrix (m) protein modulates m oligomerization and virus production. J. Virol..

[28] Li D., Jans D.A., Bardin P.G., Meanger J., Mills J., Ghildyal R. (2008). Association of respiratory syncytial virus m protein with viral nucleocapsids is mediated by the m2-1 protein. J. Virol..

[29] Kiss G., Holl J.M., Williams G.M., Alonas E., Vanover D., Lifland A.W., Gudheti M., Guerrero-Ferreira R.C., Nair V., Yi H. (2014). Structural analysis of respiratory syncytial virus reveals the position of m2-1 between the matrix protein and the ribonucleoprotein complex. J. Virol..

[30] Tawar R.G., Duquerroy S., Vonrhein C., Varela P.F., Damier-Piolle L., Castagne N., MacLellan K., Bedouelle H., Bricogne G., Bhella D. (2009). Crystal structure of a nucleocapsid-like nucleoprotein-rna complex of respiratory syncytial virus. Science.

[31] Bakker S.E., Duquerroy S., Galloux M., Loney C., Conner E., Eleouet J.F., Rey F.A., Bhella D. (2013). The respiratory syncytial virus nucleoprotein-rna complex forms a left-handed helical nucleocapsid. J. Gen. Virol..

[32] Ulloa L., Serra R., Asenjo A., Villanueva N. (1998). Interactions between cellular actin and human respiratory syncytial virus (hrsv). Virus Res..

[33] Jeffree C.E., Brown G., Aitken J., Su-Yin D.Y., Tan B.H., Sugrue R.J. (2007). Ultrastructural analysis of the interaction between f-actin and respiratory syncytial virus during virus assembly. Virology.

