# Supplementary material for: Interaction Between the Matrix Protein and the Polymerase Complex of Respiratory Syncytial Virus
Source: Viruses. 2024 Dec 4;16(12):1881. doi: 10.3390/v16121881 (PMC11680393; doi:10.3390/v16121881)
Supplement: Supplementary file 1 [file viruses-16-01881-s001.zip › viruses-3284279-supplementary.pdf]

Table S1. PCR primer sequences for cloning of L protein fragments.

Primer Name	Primer Sequence
RSV-L-frag1_F	TAA TGG ATC CGA CAT TAT TAA TGG AAA TTC
RSV-L-frag1_R	ATA AGA ATG CGG CCG CGG TTA TTG TCA CCT GCA AG
RSV-L-frag2_F	GTA GGA TCC CGG CTC AAC AAC ATC ACA GAT GC
RSV-L-frag2_R	ATA AGA ATG CGG CCG CTC GTA AGA CTT TCA GGA AAG
RSV-L-frag3_F	TAT GGA TCC GAA CCG GGA ATG TTC AGA CAG G
RSV-L-frag3_R	ATA AGA ATG CGG CCG CTG GGA TCA CCA CCA CCA AAT A
RSV-L-frag4_F	TAG GGA TCC AGT GGG ACC GTG GAT AAA CA
RSV-L-frag4_R	ATA AGA ATG CGG CCG CAT CTC TCA CCA CGT GTT AAA CTG
RSV-L-frag5_F	TCA GGA TCC ACT GGT GTT ACA TCA CCC AGT ATC A
RSV-L-frag5_R	ATA AGA ATG CGG CCG CCA CAC TCC AGC TTT GCT TTG
RSV-L-frag6_F	TCA GGA TCC AGT GAA AAA GAT TGG GGA GAG GG
RSV-L-frag6_R	ATA AGA ATG CGG CCG CAA TTC CCT GCT CCT TCA CCT ATG
RSV-L-frag7_F	TAG GGA TCC AAT CCC AAT TGT ATA GCA TTC ATA G
RSV-L-frag7_R	ATA AGA ATG CGG CCG CAT TCA TTA TGA AAG TTG TAT AAC
